# Under-prescribing of Prevention Drugs and Primary Prevention of Stroke and Transient Ischaemic Attack in UK General Practice: A Retrospective Analysis

**DOI:** 10.1371/journal.pmed.1002169

**Published:** 2016-11-15

**Authors:** Grace M. Turner, Melanie Calvert, Max G. Feltham, Ronan Ryan, David Fitzmaurice, K. K. Cheng, Tom Marshall

**Affiliations:** Institute of Applied Health Research, University of Birmingham, Birmingham, United Kingdom; University of Oxford, UNITED KINGDOM

## Abstract

**Background:**

Stroke is a leading cause of death and disability; worldwide it is estimated that 16.9 million people have a first stroke each year. Lipid-lowering, anticoagulant, and antihypertensive drugs can prevent strokes, but may be underused.

**Methods and Findings:**

We analysed anonymised electronic primary care records from a United Kingdom (UK) primary care database that covers approximately 6% of the UK population. Patients with first-ever stroke/transient ischaemic attack (TIA), ≥18 y, with diagnosis between 1 January 2009 and 31 December 2013, were included. Drugs were considered under-prescribed when lipid-lowering, anticoagulant, or antihypertensive drugs were clinically indicated but were not prescribed prior to the time of stroke or TIA. The proportions of strokes or TIAs with prevention drugs under-prescribed, when clinically indicated, were calculated.

In all, 29,043 stroke/TIA patients met the inclusion criteria; 17,680 had ≥1 prevention drug clinically indicated: 16,028 had lipid-lowering drugs indicated, 3,194 anticoagulant drugs, and 7,008 antihypertensive drugs. At least one prevention drug was not prescribed when clinically indicated in 54% (9,579/17,680) of stroke/TIA patients: 49% (7,836/16,028) were not prescribed lipid-lowering drugs, 52% (1,647/3,194) were not prescribed anticoagulant drugs, and 25% (1,740/7,008) were not prescribed antihypertensive drugs.

The limitations of our study are that our definition of under-prescribing of drugs for stroke/TIA prevention did not address patients’ adherence to medication or medication targets, such as blood pressure levels.

**Conclusions:**

In our study, over half of people eligible for lipid-lowering, anticoagulant, or antihypertensive drugs were not prescribed them prior to first stroke/TIA. We estimate that approximately 12,000 first strokes could potentially be prevented annually in the UK through optimal prescribing of these drugs. Improving prescription of lipid-lowering, anticoagulant, and antihypertensive drugs is important to reduce the incidence and burden of stroke and TIA.

## Abstract 2

In a retrospective analysis of primary care data from the United Kingdom, Grace Turner and colleagues study the proportion of patients with a stroke or transient ischemic attack who had been prescribed the clinically indicated lipid lowering, antihypertensive or anticoagulant drugs.

## Author Summary

**Why Was This Study Done?:**

- Atrial fibrillation, dyslipidaemia, and hypertension are risk factors for stroke and transient ischaemic attack (TIA). However, anticoagulant, lipid-lowering, and antihypertensive drugs have been found to reduce the incidence of stroke.
- Prescribing of these drugs for primary stroke/TIA prevention may be suboptimal in general practice. The proportion of stroke/TIA patients not prescribed prevention drugs when anticoagulant, lipid-lowering, or antihypertensive drugs are clinically indicated is unclear.

**What Did the Researchers Do and Find?:**

- We conducted a retrospective analysis of the electronic primary care medical records of 29,043 stroke/TIA patients in the UK.
- Half of the patients with clinical indications for anticoagulant drugs (52%; 1,647/3,194) or lipid-lowering drugs (49%; 7,836/16,028) were not prescribed these drugs prior to stroke/TIA. A quarter of the patients with clinical indications for antihypertensive drugs (25%; 1,740/7,008) were not prescribed these drugs prior to stroke/TIA.
- There was no reduction in the proportion of stroke/TIA patients with prior missed opportunities for prevention with lipid-lowering or antihypertensive drugs between 2009 and 2013; however, prescribing of anticoagulant drugs improved during this period.

**What Do These Findings Mean?:**

- Improving prescribing of anticoagulant, lipid-lowering, and antihypertensive drugs in primary care could potentially prevent approximately 12,000 first strokes annually in the United Kingdom.

## Introduction

Stroke is a leading cause of death and disability worldwide, with an estimated annual incidence of 16.9 million first strokes and 6 million stroke-related deaths [1]. Although the age-standardised incidence rates have decreased over the past two decades, the absolute numbers of strokes and stroke-related deaths and disability cases have increased due to the ageing population [1]. Furthermore, transient ischaemic attack (TIA) is an important risk factor for stroke that also has a high prevalence worldwide [2].

Primary prevention through treatment of modifiable risk factors [3–5] can reduce the global burden of stroke and TIA. Dyslipidaemia, atrial fibrillation, and hypertension are important modifiable risk factors for these conditions; lipid-lowering, anticoagulant, and antihypertensive drugs, respectively, have been shown to be effective at reducing stroke incidence in patients with these conditions [6–10]. Evidence-based guidelines recommend lipid-lowering drugs for people with existing cardiovascular disease (CVD) and those at high CVD risk [3]; anticoagulant drugs are recommended for patients with atrial fibrillation at high stroke risk [5]; and antihypertensive drugs are recommended for people with high blood pressure (blood pressure ≥ 160/100 mm Hg) and for people with moderately high blood pressure (blood pressure ≥ 140/90 mm Hg) who have existing CVD or are at high CVD risk [4].

Despite evidence-based guidelines, prescribing of lipid-lowering, anticoagulant, and antihypertensive drugs for primary stroke and TIA prevention may be suboptimal in primary care [11–19]. Our objective was to determine, in a large primary care database covering approximately 6% of the United Kingdom (UK) population, the proportion of people eligible for primary prevention with lipid-lowering, anticoagulant, and antihypertensive drugs but not prescribed these drugs prior to stroke or TIA.

## Methods

The full protocol for this study has been published [20]; methods are summarised in brief below. Analysis of The Health Improvement Network (THIN) database has ethical approval from the National Health Service South-East Multicentre Research Ethics Committee, subject to independent scientific review [21]. This study had approval by a scientific review committee that is administered by IMS Health Real-World Evidence Solutions (reference: 13–023).

### Study Design and Data Source

The study analysed routine electronic primary care medical records from the THIN database [22]. This is a large database of anonymised UK electronic primary care records extracted from general practices using Vision patient record software. Data within THIN are representative of the UK population, and recording of stroke and TIA in THIN has been shown to have a high positive predictive value [23]. Furthermore, Vision software is used to print prescriptions, and these are automatically retained in patients’ electronic records; therefore, prescribing data are comprehensive and accurate [24]. The database covers approximately 6% of the UK population, including 3.6 million current patients and 8.8 million former or deceased patients [25].

### Population

We defined primary stroke prevention as prevention of stroke in individuals with no prior history of stroke; therefore; the study population comprised patients with a diagnosis of first stroke, first TIA, or stroke with previous TIA. Patients were included who had a stroke/TIA diagnosis between 1 January 2009 and 31 December 2013 and were aged 18 y and over at the time of their diagnosis. The date of first-ever stroke or TIA was taken as the index date. To ensure data quality and that important patient outcomes were being recorded consistently, the index dates had to occur at least 1 y after the practice began using Vision patient record software and after the practice date of acceptable mortality recording [26]. Only patients registered at a practice for at least 1 y were included, to allow sufficient time for risk factor data to be recorded.

### Outcomes

Under-prescribing of prevention drugs was defined as people with clinical indications for lipid-lowering, anticoagulant, or antihypertensive drugs not being prescribed these drugs prior to the time of their stroke/TIA. The most recent risk factor data prior to patients’ stroke or TIA were used to determine if stroke prevention drugs were clinically indicated. Under-prescribing of prevention drugs was recorded when patients in whom a lipid-lowering or antihypertensive drug was clinically indicated had no record of a prescription for up to 90 d before their stroke or TIA (the usual maximum prescription length in the UK) and no clinical code to indicate that the patient was on these drugs. If an anticoagulant drug was clinically indicated, under-prescribing was defined as no prescription up to 120 d before the event (to allow for referral to an anticoagulation clinic) and no clinical code to indicate that the patient was on anticoagulant drugs.

Clinical indications for lipid-lowering, anticoagulant, and antihypertensive drugs were based on UK national guidelines used during the study period [4,27,28]. Lipid-lowering drugs were clinically indicated if patients had coronary heart disease (CHD), chronic kidney disease (CKD), peripheral arterial disease (PAD), TIA (in stroke patients with prior TIA), diabetes mellitus and age over 40 y, familial hypercholesterolaemia, or a 10-y CVD risk of ≥20% (Table 1). Familial hypercholesterolaemia was defined as having a clinical code for the diagnosis or total cholesterol ≥ 9 mmol/l [14]. Ten-year CVD risk was estimated using the adjusted Framingham CVD risk score, which, for consistency, was calculated 1 d prior to the index date.

**Table 1:** Clinical indications for lipid-lowering, anticoagulant, and antihypertensive drugs.

Prevention Drug	Clinical Indications for Prevention Drug	Definition of Variables
**Lipid-lowering drugs**	CHD	Presence of clinical code recorded before stroke/TIA
	CKD	Presence of clinical code recorded before stroke/TIA
	PAD	Presence of clinical code recorded before stroke/TIA
	TIA	Presence of clinical code recorded before stroke
	Diabetes mellitus and age over 40 y	Presence of clinical code recorded before stroke/TIA; age at time of stroke/TIA
	Familial hypercholesterolaemia	Presence of clinical code recorded before stroke/TIA or total cholesterol ≥ 9 mmol/l (most recent value recorded before stroke/TIA)
	10-y CVD risk of ≥20%	Framingham CVD risk score*
**Anticoagulant drugs**	Atrial fibrillation and CHADS2 score ≥ 1	Presence of clinical code recorded before stroke/TI; CHADS2 score*
**Antihypertensive drugs**	Blood pressure ≥ 160/100 mm Hg	The mean of the three most recent systolic and diastolic blood pressure recordings within 3 y prior to stroke/TIA
	Blood pressure ≥ 140/90 mm Hg and CHD, CKD, PAD, TIA, diabetes and age > 40 y, or a 10-y CVD risk of ≥20%	The mean of the three most recent systolic and diastolic blood pressure recordings within 3 y prior to stroke/TIA; presence of clinical code recorded before stroke/TIA; Framingham CVD risk score*

*Calculated 1 d prior to the index date.

CHD, coronary heart disease; CKD, chronic kidney disease; CVD, cardiovascular disease; PAD, peripheral arterial disease; TIA, transient ischaemic attack.

Anticoagulant drugs were clinically indicated if patients had a diagnosis of atrial fibrillation and were at high risk of stroke (CHADS2 score ≥ 1) (Table 1). Similar to the Framingham CVD risk score, CHADS2 scores were calculated 1 d prior to the index date. The 2006 atrial fibrillation guidelines allow a prescription of aspirin in patients with a CHADS2 score of 1 [27]. However, during the study period, important studies were published that showed aspirin to be ineffective for stroke prevention [7,29], and this recommendation was superseded in the 2014 guidelines [5]. Therefore, under-prescribing of anticoagulant drugs was based on adherence to best evidence available rather than guideline adherence.

Antihypertensive drugs were clinically indicated if patients had high blood pressure (≥160/100 mm Hg) or if patients had moderately high blood pressure (≥140/90 mm Hg) and CHD, CKD, PAD, TIA (in stroke patients with prior TIA), diabetes and age over 40 y, or a 10-y CVD risk of ≥20% (Table 1). The guidelines refer to a “sustained” blood pressure ≥160/100 mm Hg or ≥140/90 mm Hg; therefore, blood pressure was the mean of the three most recent systolic and diastolic blood pressure recordings within 3 y prior to stroke/TIA. People without three blood pressure recordings within 3 y were not included in this analysis. Patients with a clinical code to indicate a diagnosis of hypertension but whose average blood pressure recordings were lower than the thresholds given above were excluded from the analysis for antihypertensive drugs; therefore, our analyses focused on uncontrolled hypertension.

### Definitions of Variables

A comprehensive list of clinical codes (Read codes) [30] for stroke and TIA was used to identify the study cohort. Patients with a clinical code indicating history of stroke or TIA recorded before a clinical code for stroke or TIA were excluded as their true index date could not be identified. Diagnoses of atrial fibrillation, diabetes, CVD, and other comorbidities were defined by the standard list of clinical codes used to identify chronic diseases for the UK chronic disease monitoring programme (Quality and Outcomes Framework [QOF] business rules version 27 [31]), and, where present, “history of” or “resolved” clinical codes were extracted. Drug prescriptions corresponding to British National Formulary (version 67) chapters [32] for lipid-lowering, anticoagulant, and antihypertensive drugs and clinical codes indicating that the patient was on these drugs were extracted to identify treated patients. Clinical codes indicating that prevention drugs were declined or contraindicated, that a patient had white coat hypertension (for patients in whom antihypertensive drugs were clinically indicated), or that there was an adverse reaction were also extracted. Rurality (urban/rural) and Townsend deprivation quintiles were extracted for each patient [33].

### Quality Checks, Missing Data, and Extreme Values

Quality checks on THIN data are completed by the company that owns THIN, IMS Health, before data are made available for researchers [34]. Clinically implausible values were excluded for blood pressure, height, weight, body mass index, total cholesterol, and high-density lipoprotein cholesterol based on prespecified cutoff values (S1 Table). If no clinically plausible values were recorded at any time prior to the index date, the variable was categorised as missing. Absence of a clinical code for an individual diagnosis prior to the index date was taken to indicate that the diagnosis was not present at the index date. Missing data for other variables were categorised as missing. Data were initially extracted for diagnoses between 1 January 2000 and 31 December 2013; however, the number of incident stroke and TIA events recorded before 2008 was less than 15% of recorded stroke and TIA incidence after 2009 (S1 Fig). After 2009, stroke and TIA incidence were more stable; therefore, only stroke and TIA diagnoses that occurred from 1 January 2009 to 31 December 2013 were included.

### Analysis

All analysis was conducted using STATA version 12 (StataCorp). Patients were categorised as having a stroke, TIA, or stroke with previous TIA. The proportions of patients with lipid-lowering, anticoagulant, and antihypertensive drug therapy not prescribed when clinically indicated were calculated for each diagnosis category, and the difference between groups tested using Pearson’s chi-squared test. The proportions of patients with multiple drugs not prescribed were calculated. In addition, of the patients under-prescribed stroke/TIA prevention drugs, the proportions of patients previously prescribed prevention drugs but whose prescriptions had stopped were identified. Exploratory analyses (see S1 Appendix) excluded CVD risk factors and variables used to calculate Framingham and CHADS2 scores that were recorded within 1 wk prior to the index date. In addition, exploratory analysis investigated the effect of using the CHA_2_DS_2_-VASc stroke risk score and QRISK2-2014 CVD risk equation, to reflect the updated recommendations of the 2014 atrial fibrillation and lipid modification guidelines, respectively [3,5]. Exploratory analyses examined the effect of changing the 10-y CVD risk from 20% to 10%, which was also an updated recommendation from the lipid modification guidelines [3]. To reflect the 2006 guideline recommendations for atrial fibrillation, exploratory analysis investigated the use of aspirin for stroke prevention in atrial fibrillation patients with a CHADS2 score of 1 [27]. In exploratory analyses, we calculated crude estimates of under-prescribing of prevention drugs and the potential number of strokes that could be prevented each year in the UK. Detailed calculations are presented in S1 Appendix.

## Results

During the study period, 29,043 people with stroke or TIA met the inclusion criteria (16,245 stroke only, 10,446 TIA only, and 2,352 stroke with previous TIA). The median age was 74 y (interquartile range 64, 82), and 51% were female. At the time of their stroke or TIA, 17,680 patients (61%) had a clinical indication for one or more stroke prevention drugs: 9,953 had one prevention drug indicated, 6,904 had two indicated, and 823 had three indicated. In all, 16,028 (55%) patients had a clinical indication for lipid-lowering drugs, 3,194 (11%), for anticoagulant drugs, and 7,008 (24%), for antihypertensive drugs. Descriptive characteristics of patients with clinical indications for each prevention drug are presented in Table 2. A clinical code indicating that prevention drugs were declined or contraindicated, that a patient had white coat hypertension (for hypertensive patients), or that there was an adverse reaction was recorded in only 5% (869/16,028) of patients with a clinical indication for lipid-lowering drugs, 7% (244/3,194), for anticoagulant drugs, and 0.7% (47/7,008), for antihypertensive drugs (S2 Fig).

**Table 2:** Descriptive characteristics of patients with and without lipid-lowering, anticoagulant, and antihypertensive drugs clinically indicated at the time of their stroke or transient ischaemic attack.

Characteristic	Subcategory	Lipid-Lowering Drugs Clinically Indicated		Anticoagulant Drugs Clinically Indicated		Antihypertensive Drugs Clinically Indicated	
		Yes	No	Yes	No	Yes	No
**Diagnosis**	Stroke only	8,464 (52.8)	7,781 (59.8)	1,881 (58.9)	14,364 (55.6)	3,843 (54.8)	12,402 (56.3)
	TIA only	5,212 (32.5)	5,234 (40.2)	958 (30.0)	9,488 (36.7)	2,253 (32.2)	8,193 (37.2)
	Stroke with previous TIA	2,352 (14.7)	0 (0.0)	355 (11.1)	1,997 (7.7)	912 (13.0)	1,440 (6.5)
	Total	16,028 (100)	13,015 (100)	3,194 (100.0)	25,849 (100.0)	7,008 (100.0)	22,035 (100.0)
**Age (years)**	<45	115 (0.7)	997 (7.7)	6 (0.2)	1,106 (4.3)	69 (1.0)	1,043 (4.7)
	45–49	221 (1.4)	723 (5.6)	9 (0.3)	935 (3.6)	109 (1.5)	835 (3.8)
	50–54	494 (3.0)	919 (7.0)	15 (0.5)	1,398 (5.4)	190 (2.7)	1,223 (5.6)
	55–59	811 (5.1)	954 (7.3)	39 (1.2)	1,726 (6.7)	311 (4.4)	1,454 (6.6)
	60–64	1,489 (9.3)	1,097 (8.4)	70 (2.2)	2,516 (9.7)	556 (7.9)	2,030 (9.2)
	65–69	2,049 (12.8)	1,193 (9.2)	170 (5.3)	3,072 (11.9)	832 (11.9)	2,410 (10.9)
	70–74	2,638 (16.5)	1,083 (8.3)	291 (9.1)	3,430 (13.3)	1,091 (15.6)	2,630 (11.9)
	75–79	2,329 (14.5)	2,069 (15.9)	604 (18.9)	3,794 (14.7)	1,133 (16.2)	3,265 (14.8)
	80–84	2,514 (15.7)	1,791 (13.8)	760 (23.8)	3,545 (13.7)	1,179 (16.8)	3,126 (14.2)
	85–89	2,068 (12.9)	1,293 (9.9)	719 (22.5)	2,642 (10.2)	974 (13.9)	2,387 (10.8)
	90–94	1,012 (6.3)	665 (5.1)	399 (12.5)	1,278 (4.9)	453 (6.5)	1,224 (5.6)
	≥95	288 (1.8)	231 (1.8)	112 (3.5)	407 (1.6)	111 (1.6)	408 (1.9)
**Sex**	Male	8,941 (55.8)	5,263 (40.4)	1,469 (46.0)	12,735 (49.3)	3,440 (49.1)	10,764 (48.8)
	Female	7,087 (44.2)	7,752 (59.6)	1,725 (54.0)	13,114 (50.7)	3,568 (50.9)	11,271 (51.2)
BMI*	Healthy	4,655 (29.1)	4,548 (34.9)	1,108 (34.7)	8,095 (31.3)	1,953 (27.9)	7,250 (32.9)
	Underweight	339 (2.1)	373 (2.9)	98 (3.1)	614 (2.4)	135 (1.9)	577 (2.6)
	Overweight	5,995 (37.4)	4,293 (33.0)	1,141 (35.7)	9,147 (35. 4)	2,599 (37.1)	7,689 (34.9)
	Obese	4,172 (26.0)	2,442 (18.8)	651 (20.4)	5,963 (23.0)	2,010 (28.7)	4,604 (20.9)
	Missing	867 (5.4)	1,359 (10.4)	196 (6.1)	2,030 (7.9)	311 (4.4)	1,915 (8.7)
**Smoking status**	Non-smoker	3,927 (24.5)	2,410 (18.5)	886 (27.7)	5,452 (21.1)	1,626 (23.2)	4,712 (21.4)
	Ex-smoker	7,910 (49.0)	7,180 (55.2)	1,865 (58.4)	13,173 (51.0)	3,702 (52.8)	11,336 (51.4)
	Current smoker	3,716 (23.3)	2,521 (19.4)	335 (10.5)	5,916 (22.9)	1,487 (21.2)	4,764 (21.6)
	Missing	475 (3.2)	904 (6.9)	108 (3.4)	1,308 (5.1)	193 (2.8)	1,223 (5.6)
**Rurality**	Urban	5,997 (37.4)	4,881 (37.5)	1,236 (38.7)	9,642 (37.3)	2,555 (36.5)	8,323 (37.8)
	Rural	10,021 (62.5)	8,128 (62.5)	1,957 (61.3)	16,192 (62.6)	4,451 (63.5)	13,698 (62.1)
	Missing	10 (0.1)	6 (0.0)	1 (0. 0)	15 (0.1)	2 (0.0)	14 (0.1)
**Townsend deprivation quintile**	1 (least deprived)	3,709 (23.2)	3,242 (24.9)	815 (25.5)	6,136 (23.7)	1,630 (23.3)	5,321 (24.1)
	2	3,497 (21.8)	3,085 (23.7)	763 (23.9)	5,819 (22.5)	1,582 (22.6)	5,000 (22.7)
	3	3,210 (20.0)	2,685 (20.6)	670 (21.0)	5,225 (20.2)	1,405 (20.0)	4,490 (20.4)
	4	3,047 (19.0)	2,201 (16.9)	528 (16.5)	4,720 (18.3)	1,323 (18.9)	3,925 (17.8)
	5 (most deprived)	2,187 (13.6)	1,486 (11.4)	347 (10.9)	3,326 (12.9)	900 (12.8)	2,773 (12.6)
	Missing	378 (2.4)	316 (2.5)	71 (2.2)	623 (2.4)	168 (2.4)	526 (2.4)
**Comorbidity**	Atrial fibrillation	2,392 (14.9)	1,152 (8.9)	3,194 (100. 0)	350 (1.4)	923 (13.2)	2,621 (11.9)
	Asthma	1,724 (10.8)	1,338 (10.3)	320 (10.0)	2,742 (10.6)	736 (10.5)	2,326 (10.6)
	Cancer	1,911 (11.9)	1,328 (10.2)	420 (13.1)	2,819 (10.9)	796 (11.4)	2,443 (11.1)
	CHD	5,543 (34.6)	0 (0.0)	1,083 (33.9)	4,460 (17.3)	2,023 (28.9)	3,520 (16.0)
	CKD	5,774 (36.0)	0 (0.0)	1,157 (36.2)	4,617 (17.9)	2,343 (33.4)	3,431 (15.6)
	COPD	1,470 (9.2)	728 (5.6)	309 (9.7)	1,889 (7.3)	547 (7.8)	1,651 (7.5)
	Dementia	737 (4.6)	533 (4.1)	213 (6.7)	1,057 (4.1)	226 (3.2)	1,044 (4.7)
	Depression	3,420 (21.3)	2,754 (21.2)	613 (19.2)	5,561 (21.5)	1,413 (20.2)	4,761 (21.6)
	Diabetes	4,486 (28.0)	26 (0.2)	658 (20.6)	3,854 (14.9)	1,796 (25.6)	2,716 (12.3)
	Epilepsy	287 (1.8)	327 (2.5)	47 (1.5)	567 (2.2)	117 (1.7)	497 (2.3)
	Heart failure	1,338 (8.3)	287 (2.2)	651 (20.4)	974 (3.8)	437 (6.2)	1,188 (5.4)
	Hypertension	9,666 (60.3)	4,980 (38.3)	2,297 (71.9)	12,349 (47.8)	5,241 (74.8)	9,405 (42.7)
	Hypothyroidism	1,724 (10.8)	1,166 (9.0)	440 (13.8)	2,450 (9.5)	755 (10.8)	2,135 (9.7)
	Learning disability	54 (0.3)	76 (0.6)	6 (0.2)	124 (0.5)	16 (0.2)	114 (0.5)
	Osteoporosis	1,265 (7.9)	1,053 (8.1)	372 (11.6)	1,946 (7.5)	578 (8.2)	1,740 (7.9)
	PAD	1,431 (8.9)	0 (0.0)	216 (6.8)	1,215 (4.7)	576 (8.2)	855 (3.9)
	Palliative care	223 (1.4)	136 (1.0)	52 (1.6)	307 (1.2)	67 (1.0)	292 (1.3)
	Psychosis	262 (1.6)	177 (1.4)	30 (0.9)	409 (1.6)	96 (1.4)	343 (1.6)
	Rheumatoid arthritis	394 (2.5)	261 (2.0)	80 (2.5)	575 (2.2)	170 (2.4)	485 (2.2)

Data are given as frequency (percent).

*BMI: healthy (18.5–25.9 kg/m^2^), underweight (<18.5 kg/m^2^), overweight (26–30 kg/m^2^), obese (>30 kg/m^2^)

BMI, body mass index; CHD, coronary heart disease; CKD, chronic kidney disease; COPD, chronic obstructive pulmonary disease; PAD, peripheral artery disease; TIA, transient ischaemic attack.

### Under-prescribing of Drugs for Stroke/TIA Prevention

Fifty-four percent (9,579/17,680) of people with a clinical indication for one or more prevention drugs prior to stroke or TIA were not prescribed these drugs; in the majority of these cases, one drug was not prescribed (83%; 7,969/9,579), in 16% (1,576/9,579) two drugs were not prescribed, and in 0.4% (34/9,579) three drugs were not prescribed (Table 3). The combinations of multiple prevention drugs under-prescribed are presented in S2 Fig. Under-prescribing of prevention drugs was found in 49% (7,836/16,028) of patients with a clinical indication for lipid-lowering drugs, 52% (1,647/3,194), for anticoagulant drugs, and 25% (1,740/7,008), for antihypertensive drugs (Table 4). There was no significant difference in the proportion of people with antihypertensive drugs under-prescribed among patients with stroke only, TIA only, or stroke with previous TIA (*p* = 0.21; odds ratio [OR] 0.91, 95% CI 0.81–1.02, for stroke versus TIA; OR 1.04, 95% CI 0.89–1.23, for stroke versus stroke with previous TIA). However, for the other two classes of prevention drugs, there was a significant difference between patients with stroke only, TIA only, or stroke with previous TIA: lipid-lowering drug prescribing, *p* < 0.01 (OR 0.83, 95% CI 0.77–0.89, for stroke versus TIA; OR 0.97, 95% CI 0.89–1.07, for stroke versus stroke with previous TIA), and anticoagulant drug prescribing, *p* = 0.02 (OR 1.02, 95% CI 0.88–1.12, for stroke versus TIA; OR 0.73, 95% CI 0.58–0.92, for stroke versus stroke with previous TIA) (Table 4). Exploratory analysis excluded CVD risk factors and variables used to calculate Framingham and CHADS2 scores that were recorded within 1 wk prior to the index date. There was minimal difference in the proportion of under-prescribing of prevention drugs with this exclusion criterion: anticoagulant drugs, 51.1% (1,597/3,123); lipid-lowering drugs, 48.7% (7,767/15,945); and antihypertensive drugs, 24.3% (1,677/6,899).

**Table 3:** Proportion of stroke and transient ischaemic attack patients under-prescribed one, two, or three prevention drugs (lipid-lowering, anticoagulant, or antihypertensive drugs).

Number of Prevention Drugs Not Prescribed When Clinically Indicated	Proportion of Stroke/TIA Patients Not Prescribed Prevention Drugs When Clinically Indicated, Percent (Frequency)*
1	83.2 (7,969/9,579)
2	16.4 (1,576/9,579)
3	0.4 (34/9,579)

*Number of people with prevention drugs not prescribed divided by the number of people eligible for one or more prevention drug.

TIA, transient ischaemic attack.

**Table 4:** Proportion of stroke and transient ischaemic attack patients under-prescribed lipid-lowering, anticoagulant, and antihypertensive drugs for primary prevention.

Diagnosis	Proportion of Strokes/TIAs with Prevention Drugs Not Prescribed, Percent (Frequency)*		
	Lipid-Lowering drugs	Anticoagulant Drugs	Antihypertensive Drugs
Stroke	50.5 (4,276/8,464)	52.3 (983/1,881)	25.3 (971/3,843)
TIA	45.8 (2,387/5,212)	52.8 (506/958)	23.6 (531/2,253)
Stroke with previous TIA	49.9 (1,173/2,352)	44.5 (158/355)	26.1 (238/912)
Total	48.9 (7,836/16,028)	51.6 (1,647/3,194)	24.8 (1,740/7,008)

*Number of people with prevention drugs not prescribed divided by the number of people eligible for each prevention drug.

TIA, transient ischaemic attack.

### Change over Time

**Fig 1:**
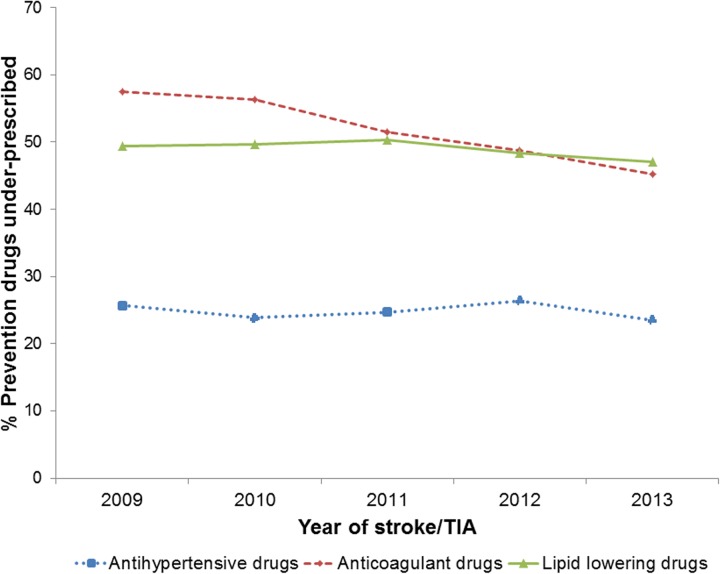
Under-prescribing of lipid-lowering, anticoagulant, and antihypertensive drugs between 2009 and 2013 in patients prior to stroke or transient ischaemic attack. TIA, transient ischaemic attack.

There was a marked decrease in the under-prescribing of anticoagulant drugs between 2009 (58%) and 2013 (45%), but this was not observed for lipid-lowering and antihypertensive drug prescribing (Fig 1).

### Prescribing Stopped versus Never Prescribed

**Fig 2:**
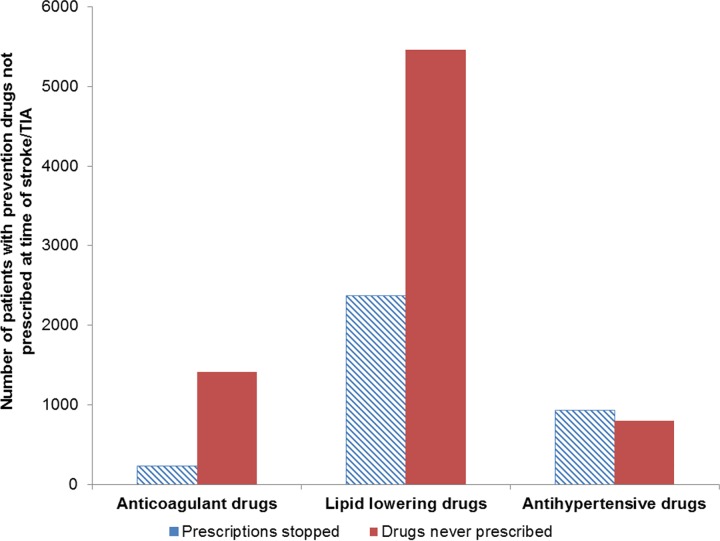
Comparison of patients who were previously prescribed prevention drugs but whose prescriptions had stopped at the time of stroke/transient ischaemic attack and patients who had no history of prescriptions. TIA, transient ischaemic attack.

Of the patients who were not prescribed stroke/TIA prevention drugs when clinically indicated, the proportion of patients who had been previously prescribed a prevention drug but whose prescription had stopped at the time of stroke/TIA was 14% (235/1,647) for anticoagulant drugs, 30% (2,350/7,836) for lipid-lowering drugs, and 54% (938/1,740) for antihypertensive drugs (Fig 2). Results of additional exploratory analyses are presented in S1 Appendix.

### Estimates of Under-prescribing of Prevention Drugs in the UK Population

Extrapolating the proportions of underuse of prevention drugs within the THIN database to estimates of the UK population and stroke incidence, we estimate that 41,405 first stroke patients are eligible for but not prescribed lipid-lowering, anticoagulant, or antihypertensive drugs annually. Based on the effectiveness of statins, anticoagulant, and antihypertensive drugs, the number of strokes that could potentially be prevented each year in the UK by optimal prescribing is estimated to be 11,823 (Table 5).

**Table 5:** Estimated annual incidence of stroke and number of strokes that could potentially be prevented annually in the UK.

Age Band	Number of Strokes per Year in UK		Estimated Number of Strokes That Could Be Prevented in UK	
	Male	Female	Male	Female
<35 y	0	0	0	0
35–44 y	1,469	896	50	30
45–54 y	2,453	1,097	184	46
55–64 y	6,712	4,413	784	256
65–74 y	18,817	12,744	2,426	1,063
75–84 y	14,656	20,001	1,288	1,931
≥85 y	9,747	16,677	1,237	2,529

## Discussion

In this study carried out in UK primary care, six out of ten patients who had a first stroke or TIA were eligible for at least one prevention drug at the time of their stroke or TIA; over half of these were not prescribed prevention drugs that were clinically indicated. In effect, one-third of all strokes or TIAs occurred in patients who had prevention drugs clinically indicated but were not prescribed them. This included half of patients who had lipid-lowering or anticoagulant drugs clinically indicated and a quarter of patients who had antihypertensive drugs clinically indicated. The under-prescribing of anticoagulants decreased between 2009 and 2013, but there was no change for lipid-lowering and antihypertensive drugs. Over half of the patients not prescribed antihypertensive drugs when clinically indicated had previously been prescribed these drugs, but their prescriptions had stopped, compared to only 14% for anticoagulant drugs and 30% for lipid-lowering drugs. Our findings indicate underuse of lipid-lowering, anticoagulant, and antihypertensive drugs in UK primary care in patients for whom these drugs are clinically indicated for prevention of stroke or TIA.

The strengths of this study are that the dataset is representative of UK general practice and the data are recent. The prescribing data are accurate and comprehensively recorded [23], and the sample size is very large. Stroke and the main comorbidities are likely to be accurately recorded as they are clinically significant, diagnoses have been validated within THIN [22], and, in the UK, general practitioners (GPs) are incentivised to keep a register of patients with these conditions. However, restricting the definition of comorbidities to QOF clinical codes may result in diagnoses being missed if they were recorded using alternative clinical codes. This was an epidemiological, descriptive study; therefore, an important limitation is that the reasons for non-prescribing are unclear. There may be legitimate reasons why patients were not prescribed prevention drugs that were not available in our dataset, such as bleeding risk when prescribing anticoagulant drugs or knowledge of a patient’s adherence to medication. Clinical codes indicating that prevention drugs were declined or contraindicated or that there was an adverse reaction were extracted. However, patients with these clinical codes recorded were not excluded from the analysis because it is unclear if these were currently relevant or historic codes; we note that the number of patients in our sample with these codes was small (5%, 7%, and 0.7% for lipid-lowering, anticoagulant, and antihypertensive drugs, respectively). Furthermore, prevention of stroke/TIA is complex, and our definition of under-prescribing does not address patients’ adherence to medication, appropriate prescribing of drug combinations, or medication targets, such as blood pressure levels. We defined under-prescribing of anticoagulant drugs as no prescription of these drugs to atrial fibrillation patients with a CHADS2 score of ≥1 prior to stroke or TIA. Under-prescribing of anticoagulants based on adherence to the 2006 guidelines allowing prescription of aspirin in patients with a CHADS2 score of 1 [27] were explored in a sensitivity analysis (see S1 Appendix). Exploratory analysis also investigated the impact of updated guidelines regarding use of the CHA_2_DS_2_-VASc and QRISK2-2014 risk scores to reflect guideline updates [3,5] (see S1 Appendix). Finally, the extrapolations of the findings to the UK population to determine the extent of under-prescribing of prevention drugs and the potential number of strokes that could be prevented in the UK are crude estimates. These analyses are intended to highlight the scale and impact of under-prescribing of stroke prevention drugs rather than to provide precise estimates.

Using estimates of the UK population [35], stroke incidence [36], and the effectiveness of statins, anticoagulants, and antihypertensive drugs [6–10], we determined the extent of under-prescribing of primary stroke prevention drugs in the UK (see Table E in S1 Appendix). We estimate that approximately 41,400 first stroke patients are eligible for but not prescribed lipid-lowering, anticoagulant, or antihypertensive drugs annually. Based on the relative risk reduction of these drugs, approximately 12,000 strokes could potentially be prevented each year in the UK by optimal prescribing of stroke prevention drugs (see Table F in S1 Appendix). These estimates demonstrate the potential impact of improving prescription of primary stroke prevention drugs in the UK. Given that stroke is the second leading cause of death and third leading cause of disability-adjusted life-years lost worldwide [37,38], improving primary stroke and TIA prevention is important to reduce the incidence and burden of these conditions.

Of the three prevention drugs, lipid-lowering drugs were the most commonly clinically indicated; over half of the people who had a stroke or TIA were eligible for these drugs. Statin drugs account for the majority of lipid-lowering drugs prescribed [39], and prescribing has increased over the past decade [40]; nevertheless, there is controversy regarding administration of these drugs for primary stroke prevention. Fears about side effects [41], polypharmacy [41], and the medicalisation of “healthy” patients [42] have been identified as barriers to GPs prescribing statins. However, statins are effective at reducing the incidence of stroke [9], and statin-induced side effects are likely to be less frequent than originally thought [43]. These drugs are often more commonly associated with CHD prevention; however, our findings demonstrate the potential impact of improving prescription of lipid-lowering drugs in the context of stroke prevention. Importantly, under-prescribing of lipid-lowering drugs for primary stroke prevention is likely to rise given the most recent guideline recommendations, which increase the number of people eligible for these drugs (see S1 Appendix) [3]. Furthermore, in the UK, QOF introduced an incentive for statin prescribing for primary CVD prevention as recently as 2013, and only patients aged 35 to 74 y with a new diagnosis of hypertension and CVD risk ≥20% are eligible [31].

The proportion of stroke/TIA patients with anticoagulant drugs under-prescribed decreased in the relatively short time period of the study (58% in 2009 to 45% in 2013), but still remained substantial. During this period, there were subtle changes in the UK incentives for anticoagulant prescribing: from 2009 to 2011, QOF incentivised either anticoagulants or antiplatelet agents for patients with atrial fibrillation (regardless of stroke risk); in 2011/2012, stroke risk was introduced (anticoagulants/antiplatelet agents for people with atrial fibrillation and a CHADS2 score of 1, anticoagulants for people with atrial fibrillation and CHADS2 score > 1) [31]. Older age has been reported by clinicians as one of the main reasons for not prescribing anticoagulants [44]. This is particularly relevant because atrial fibrillation is more prevalent in the elderly, stroke risk in atrial fibrillation increases in the elderly (median age of patents with anticoagulants indicated was 82 y), and the population is ageing [45,46]. Bleeding risk, falls risk, and polypharmacy, particularly in those with a reduced life expectancy, are likely to be reasons for reduced prescribing in the elderly [44]. However, the benefits of anticoagulation in the elderly have been shown to outweigh the risks, and the net benefit of anticoagulation is actually greatest in the elderly [29]. Under-prescribing of anticoagulants for atrial fibrillation patients in particular has the potential to cause a huge burden on patients and society because strokes in these patients are associated with greater post-stroke disability and mortality [47,48].

Hypertension is one of the most well documented risk factors for stroke, and there is a well-established evidence base for the use of antihypertensive drugs for primary stroke prevention [4]; therefore, it is unclear why prescribing remains suboptimal. Of the three prevention drugs, the lowest proportion of under-prescribing was found for antihypertensive drugs. This could be a result of the strong evidence base, the safety profile, and the low cost of these drugs [4]. However, the absolute number of stroke or TIA patients with these drugs under-prescribed was higher than that for anticoagulant drugs (1,647 versus 1,740 patients for anticoagulant and antihypertensive drugs, respectively). Hypertension is a common comorbidity and contributes to over half of strokes globally [49]; therefore, under-prescribing of antihypertensive drugs affects a large number of people and is likely to have significant implications for stroke incidence.

There may be legitimate reasons for not prescribing stroke prevention drugs to people with clinical indications, and, arguably, some of the non-prescribing reported by our study may not represent missed opportunities for prevention. However, as discussed, evidence suggests GPs may overestimate side effects [43] and underestimate the benefit for elderly patients [29]. Multiple GP-related barriers to guideline adherence have been identified, including knowledge of guidelines and lack of agreement or outcome expectancy [50]. Research suggests that GPs’ recommendations highly influence patients’ preferences, which reinforces the importance of addressing GP-related barriers [51]. However, guideline adherence and prescribing behaviour is complex, and the problem is not limited to GP-related behaviours. Other barriers include patient factors, such as patient preference and understanding/perception of risk, and environmental factors, such as time and resource constraints [50]. This complexity is highlighted by our finding that there was a difference between the three prevention drugs in the proportion of prescriptions stopped compared to those never prescribed in patients with drugs under-prescribed. Therefore, it is important to understand the different behavioural mechanisms related to non-prescribing for each drug and to consider this complex behavioural system for future research and intervention development.

Under-prescribing of drugs for stroke prevention has been reported by other international studies. A survey of 66 general practices from 12 European countries found similar rates of under-prescribing (50%) of lipid-lowering drugs in people with hypercholesterolaemia; however, only 14% of people with elevated blood pressure were not prescribed blood-pressure-lowering medication [52]. Similarly, a cross-sectional study of 162 Italian GPs and 3,120 patients found that treatment levels were high for people with hypertension (96%), but low for people with hyperlipidaemia (46%) [19]. There is evidence to suggest that identification and treatment of hypertension may be higher in the United States of America and Canada than in other countries [53]. However, it is difficult to compare the prescribing rates of these studies with those of ours because different definitions of under-prescribing were used.A worldwide (30 countries) observational registry of newly diagnosed atrial fibrillation patients found that anticoagulants were prescribed in 61% of patients; however, there was overuse of these drugs in people at low risk of stroke [54].

In conclusion, our findings quantify the underuse of lipid-lowering, anticoagulant, and antihypertensive drugs for primary stroke and TIA prevention in UK primary care. Dyslipidaemia, atrial fibrillation, and hypertension are three of the most important risk factors for stroke and TIA; therefore, our finding that medical management of these conditions is inadequate has important clinical and policy implications. Substantial numbers of strokes and TIAs could potentially be prevented through improving prescription of these drugs in primary care, which would contribute to reducing the burden of these conditions.

## Supporting Information

S1 Appendix Methods and results of the exploratory analyses. (DOCX)

S1 Fig Crude incidence of stroke and transient ischaemic attack recorded in The Health Improvement Network database. (DOCX)

S2 Fig Summary of under-prescribing of stroke prevention drugs and exception reporting (dugs declined or contraindicated). (DOCX)

S1 Protocol Published protocol paper. (PDF)

S1 STROBE Checklist STROBE checklist. (DOCX)

S1 Table Values outside clinically plausible ranges, which were excluded. (DOCX)

S2 Table Number of general practices contributing data to the study between 2000 and 2013. (DOCX)

## Abbreviations

CHD: coronary heart disease
CKD: chronic kidney disease
CVD: cardiovascular disease
GP: general practitioner
OR: odds ratio
PAD: peripheral arterial disease
QOF: Quality and Outcomes Framework
THIN: The Health Improvement Network
TIA: transient ischaemic attack

## References

[1] Krishnamurthi RV, Feigin VL, Forouzanfar MH, Mensah GA, Connor M, Bennett DA, et al Global and regional burden of first-ever ischaemic and haemorrhagic stroke during 1990–2010: findings from the Global Burden of Disease Study 2010. Lancet Glob Health. 2013;1(5):e259–81. 10.1016/S2214-109X(13)70089-5 25104492 PMC4181351

[2] Sacco RL. Risk factors for TIA and TIA as a risk factor for stroke. Neurology. 2004;62(8 Suppl 6):S7–11. 15111649 10.1212/wnl.62.8_suppl_6.s7

[3] National Institute for Health and Care Excellence. Lipid modification: cardiovascular risk assessment and the modification of blood lipids for the primary and secondary prevention of cardiovascular disease Clinical Guideline 181. London: National Clinical Guideline Centre; 2014. 25340243

[4] National Institute for Health and Clinical Excellence. Hypertension: the clinical management of primary hypertension in adults Clinical Guideline 127. London: National Clinical Guideline Centre; 2011.

[5] National Institute for Health and Clinical Excellence. Atrial fibrillation: the management of atrial fibrillation Clinical Guideline 180. London: National Clinical Guideline Centre; 2014.

[6] Aguilar MI, Hart R. Oral anticoagulants for preventing stroke in patients with non-valvular atrial fibrillation and no previous history of stroke or transient ischemic attacks. Cochrane Database Syst Rev. 2005;(3):CD001927 10.1002/14651858.CD001927.pub2 16034869 PMC8408914

[7] Aguilar MI, Hart R, Pearce LA. Oral anticoagulants versus antiplatelet therapy for preventing stroke in patients with non-valvular atrial fibrillation and no history of stroke or transient ischemic attacks. Cochrane Database Syst Rev. 2007;(3):CD006186 10.1002/14651858.CD006186.pub2 17636831 PMC13591242

[8] Psaty BM, Lumley T, Furberg CD, Schellenbaum G, Pahor M, Alderman MH, et al Health outcomes associated with various antihypertensive therapies used as first-line agents: a network meta-analysis. JAMA. 2003;289(19):2534–44. 10.1001/jama.289.19.2534 12759325

[9] Taylor F, Huffman MD, Macedo AF, Moore TH, Burke M, Davey Smith G, et al Statins for the primary prevention of cardiovascular disease. Cochrane Database Syst Rev. 2013;(1):CD004816 10.1002/14651858.CD004816.pub5 23440795 PMC6481400

[10] Wang W, Zhang B. Statins for the prevention of stroke: a meta-analysis of randomized controlled trials. PLoS ONE. 2014;9(3):e92388 10.1371/journal.pone.0092388 24643199 PMC3958535

[11] Heneghan C, Perera R, Mant D, Glasziou P. Hypertension guideline recommendations in general practice: awareness, agreement, adoption, and adherence. Br J Gen Pract. 2007;57(545):948–52. 10.3399/096016407782604965 18252069 PMC2084133

[12] Joffres M, Falaschetti E, Gillespie C, Robitaille C, Loustalot F, Poulter N, et al Hypertension prevalence, awareness, treatment and control in national surveys from England, the USA and Canada, and correlation with stroke and ischaemic heart disease mortality: a cross-sectional study. BMJ Open. 2013;3(8):e003423 10.1136/bmjopen-2013-003423 23996822 PMC3758966

[13] Khatib R, Schwalm J-D, Yusuf S, Haynes RB, McKee M, Khan M, et al Patient and healthcare provider barriers to hypertension awareness, treatment and follow up: a systematic review and meta-analysis of qualitative and quantitative studies. PLoS ONE. 2014;9(1):e84238 10.1371/journal.pone.0084238 24454721 PMC3893097

[14] Koivisto P, Koivisto U, Miettinen T, Kontula K. Diagnosis of heterozygous familial hypercholesterolemia. DNA analysis complements clinical examination and analysis of serum lipid levels. Arterioscler Thromb Vasc Biol. 1992;12(5):584–92. 10.1161/01.atv.12.5.584 1315570

[15] Missault L, Witters N, Imschoot J. High cardiovascular risk and poor adherence to guidelines in 11 069 patients of middle age and older in primary care centres. Eur J Cardiovasc Prev Rehabil. 2010;17(5):593–8. 10.1097/HJR.0b013e328339cc86 20389248

[16] Ogilvie IM, Newton N, Welner SA, Cowell W, Lip GY. Underuse of oral anticoagulants in atrial fibrillation: a systematic review. Am J Med. 2010;123(7):638–45.e4. 10.1016/j.amjmed.2009.11.025 20609686

[17] Palm F, Kleemann T, Dos Santos M, Urbanek C, Buggle F, Safer A, et al Stroke due to atrial fibrillation in a population-based stroke registry (Ludwigshafen Stroke Study) CHADS2, CHA2DS2-VASc score, underuse of oral anticoagulation, and implications for preventive measures. Eur J Neurol. 2013;20(1):117–23. 10.1111/j.1468-1331.2012.03804.x 22788384

[18] Partington SL, Abid S, Teo K, Oczkowski W, O’Donnell MJ. Pre-admission warfarin use in patients with acute ischemic stroke and atrial fibrillation: the appropriate use and barriers to oral anticoagulant therapy. Thromb Res. 2007;120(5):663–9. 10.1016/j.thromres.2006.12.019 17434577

[19] Roccatagliata D, Avanzini F, Monesi L, Caimi V, Lauri D, Longoni P, et al Is global cardiovascular risk considered in current practice? Treatment and control of hypertension, hyperlipidemia, and diabetes according to patients’ risk level. Vasc Health Risk Manag. 2006;2(4):507–14. 17323606 10.2147/vhrm.2006.2.4.507 PMC1994019

[20] Moran GM, Calvert M, Feltham MG, Marshall T. Retrospective case review of missed opportunities for primary prevention of stroke and TIA in primary care: protocol paper. BMJ Open. 2014;4:e006622 10.1136/bmjopen-2014-006622 25387760 PMC4244480

[21] IMS Health. Ethics. London: IMS Health; 2015 [cited 2016 Oct 15]. Available from: http://csdmruk.cegedim.com/our-data/ethics.shtml.

[22] The Health Improvement Network. Partnership. London: CSD Health Research; 2016 [cited 2016 Oct 15]. Available from: http://www.thin-uk.com/.

[23] Ruigomez A, Martin-Merino E, Rodriguez LA. Validation of ischemic cerebrovascular diagnoses in the health improvement network (THIN). Pharmacoepidemiol Drug Saf. 2010;19(6):579–85. 10.1002/pds.1919 20131328

[24] In Practice Systems. The Health Improvement Network (THIN). London: In Practice Systems; 2016 [cited 2016 Oct 15]. Available from: http://www.inps.co.uk/vision/health-improvement-network-thin.

[25] IMS Health. Statistics. London: IMS Health; 2005 [cited 2016 Oct 15]. Available from: http://csdmruk.cegedim.com/our-data/statistics.shtml.

[26] Maguire A, Blak B, Thompson M. The importance of defining periods of complete mortality reporting for research using automated data from primary care. Pharmacoepidemiol Drug Saf. 2009;18(1):76–83. 10.1002/pds.1688 19065600

[27] National Collaborating Centre for Chronic Conditions. Atrial fibrillation: national clinical guideline for management in primary and secondary care Clinical Guideline 36. London: National Clinical Guideline Centre; 2006.

[28] National Institute for Health and Care Excellence. Lipid modification: cardiovascular risk assessment and the primary and secondary prevention of cardiovascular disease Clinical Guideline 67. London: National Clinical Guideline Centre; 2008.

[29] Mant J, Hobbs FD, Fletcher K, Roalfe A, Fitzmaurice D, Lip GY, et al Warfarin versus aspirin for stroke prevention in an elderly community population with atrial fibrillation (the Birmingham Atrial Fibrillation Treatment of the Aged Study, BAFTA): a randomised controlled trial. Lancet. 2007;370(9586):493–503. 10.1016/S0140-6736(07)61233-1 17693178

[30] Health and Social Care Information Centre. Read codes London: Health and Social Care Information Centre; 2016 [cited 2016 Oct 15]. Available from: http://systems.hscic.gov.uk/data/uktc/readcodes/index_html.

[31] Health and Social Care Information Centre. QOF business rules version 27 Leeds: Primary Care Commissioning; 2016 [cited 2016 Oct 15]. Available from: http://www.pcc-cic.org.uk/article/qof-business-rules-v27.

[32] Midlov P, Ekesbo R, Johansson L, Gerward S, Persson K, Nerbrand C, et al Barriers to adherence to hypertension guidelines among GPs in southern Sweden: a survey. Scand J Prim Health Care. 2008;26(3):154–9. 10.1080/02813430802202111 18609250 PMC3409603

[33] CSD Medical Research UK. THIN data guide for researchers London: CSD Medical Research UK; 2014. 114 p.

[34] IMS Health. Quality assurance London: IMS Health; 2015 [cited 2016 Oct 15]. Available from: http://www.epic-uk.org/our-data/data-quality.shtml.

[35] London Datastore. Office for National Statistics (ONS) population estimates, borough and ward London: London Datastore; 2016 [cited 2016 Oct 15]. Available from: http://data.london.gov.uk/dataset/office-national-statistics-ons-population-estimates-borough.

[36] Rothwell PM, Coull AJ, Silver LE, Fairhead JF, Giles MF, Lovelock CE, et al Population-based study of event-rate, incidence, case fatality, and mortality for all acute vascular events in all arterial territories (Oxford Vascular Study). Lancet. 2005;366(9499):1773–83. 10.1016/S0140-6736(05)67702-1 16298214

[37] Murray CJL, Vos T, Lozano R, Naghavi M, Flaxman AD, Michaud C, et al Disability-adjusted life years (DALYs) for 291 diseases and injuries in 21 regions, 1990–2010: a systematic analysis for the Global Burden of Disease Study 2010. Lancet. 2012;380(9859):2197–223. 10.1016/S0140-6736(12)61689-4 23245608

[38] Lozano R, Naghavi M, Foreman K, Lim S, Shibuya K, Aboyans V, et al Global and regional mortality from 235 causes of death for 20 age groups in 1990 and 2010: a systematic analysis for the Global Burden of Disease Study 2010. Lancet. 2012;380(9859):2095–128. 10.1016/S0140-6736(12)61728-0 23245604 PMC10790329

[39] National Health Service. Prescription cost analysis, England–2013 [NS] London: National Health Service; 2014 [cited 2016 Oct 15]. Available from: http://www.hscic.gov.uk/catalogue/PUB13887.

[40] United Nations Department of Economic and Social Affairs. World population prospects, the 2015 revision. United Nations Department of Economic and Social Affairs; 2015 [cited 2016 Oct 15]. Available from: http://esa.un.org/wpp/Excel-Data/population.htm.

[41] AB E, Denig P, Van Vliet T, Dekker J. Reasons of general practitioners for not prescribing lipid-lowering medication to patients with diabetes: a qualitative study. BMC Fam Pract. 2009;10(1):24. 19383116 10.1186/1471-2296-10-24 PMC2676243

[42] Kedward J, Dakin L. A qualitative study of barriers to the use of statins and the implementation of coronary heart disease prevention in primary care. Br J Gen Pract. 2003;53(494):684–9. 15103875 PMC1314690

[43] Finegold JA, Manisty CH, Goldacre B, Barron AJ, Francis DP. What proportion of symptomatic side effects in patients taking statins are genuinely caused by the drug? Systematic review of randomized placebo-controlled trials to aid individual patient choice. Eur J Prev Cardiol. 2014;21(4):464–74. 10.1177/2047487314525531 24623264

[44] Pugh D, Pugh J, Mead GE. Attitudes of physicians regarding anticoagulation for atrial fibrillation: a systematic review. Age Ageing. 2011;40(6):675–83. 10.1093/ageing/afr097 21821732

[45] Benjamin EJ, Levy D, Vaziri SM, D’Agostino RB, Belanger AJ, Wolf PA. Independent risk factors for atrial fibrillation in a population-based cohort: the Framingham Heart Study. JAMA. 1994;271(11):840–4. 8114238

[46] Chugh SS, Havmoeller R, Narayanan K, Singh D, Rienstra M, Benjamin EJ, et al Worldwide epidemiology of atrial fibrillation: a global burden of disease 2010 study. Circulation. 2013;129(8):837–47. 10.1161/CIRCULATIONAHA.113.005119 24345399 PMC4151302

[47] Lin HJ, Wolf PA, Kelly-Hayes M, Beiser AS, Kase CS, Benjamin EJ, et al Stroke severity in atrial fibrillation. The Framingham Study. Stroke. 1996;27(10):1760–4. 8841325 10.1161/01.str.27.10.1760

[48] Wolf PA, Abbott RD, Kannel WB. Atrial fibrillation as an independent risk factor for stroke: the Framingham Study. Stroke. 1991;22(8):983–8. 1866765 10.1161/01.str.22.8.983

[49] Lawes CMM, Hoorn SV, Rodgers A. Global burden of blood-pressure-related disease, 2001. Lancet. 2008;371(9623):1513–8. 10.1016/S0140-6736(08)60655-8 18456100

[50] Cabana MD, Rand CS, Powe NR, Wu AW, Wilson MH, Abboud PA, et al Why don’t physicians follow clinical practice guidelines? A framework for improvement. JAMA. 1999;282(15):1458–65. 10535437 10.1001/jama.282.15.1458

[51] Gale N, Greenfield S, Gill P, Gutridge K, Marshall T. Patient and general practitioner attitudes to taking medication to prevent cardiovascular disease after receiving detailed information on risks and benefits of treatment: a qualitative study. BMC Fam Pract. 2011;12:59 10.1186/1471-2296-12-59 21703010 PMC3135546

[52] Kotseva K, Wood D, De Backer G, De Bacquer D, Pyörälä K, Reiner Ž, et al EUROASPIRE III. Management of cardiovascular risk factors in asymptomatic high-risk patients in general practice: cross-sectional survey in 12 European countries. Eur J Cardiovasc Prev Rehabil. 2010;17(5):530–40. 10.1097/HJR.0b013e3283383f30 20577089

[53] Wolf-Maier K, Cooper RS, Kramer H, Banegas JR, Giampaoli S, Joffres MR et al Hypertension treatment and control in five European countries, Canada, and the United States. Hypertension. 2004;43:10–17. 10.1161/01.HYP.0000103630.72812.10 14638619

[54] Lip GY, Rushton-Smith SK, Goldhaber SZ, Fitzmaurice DA, Mantovani LG, Goto S, et al Does sex affect anticoagulant use for stroke prevention in nonvalvular atrial fibrillation? The prospective global anticoagulant registry in the FIELD-Atrial Fibrillation. Circ Cardiovasc Qual Outcomes. 2015;8(2 Suppl 1):S12–20. 25714828 10.1161/CIRCOUTCOMES.114.001556

