# Supplementary material for: Under-prescribing of Prevention Drugs and Primary Prevention of Stroke and Transient Ischaemic Attack in UK General Practice: A Retrospective Analysis
Source: PLoS Med. 2016 Nov 15;13(11):e1002169. doi: 10.1371/journal.pmed.1002169 (PMC5112771; doi:10.1371/journal.pmed.1002169)
Supplement: S1 Appendix — (DOCX) [file pmed.1002169.s001.docx]

S1 Appendix

**Exploratory analysis**

**Methods**

***Under-prescribing of multiple prevention drugs***

Different combinations of prevention drugs clinically indicated and not prescribed were explored.

***Under-prescribing of lipid lowering drugs***

The most recent United Kingdom (UK) dyslipidaemia guidelines (2014) lowered the definition of high CVD risk from ≥20% to ≥10% over 10 years; therefore, the effect of lowering the CVD risk threshold was explored. To also reflect the updated 2014 guidelines, where the QRISK2 risk calculation was recommended over the Framingham risk equation, sensitivity analysis investigated the impact of using QRISK2-2014 to calculate CVD risk. The difference in variables included in the Framingham and QRISK2-2014 equations are presented in Table A.

**Table A: Variables included in Framingham and QRISK2-2014 equations**

| **Variable** | **Framingham** | **QRISK2-2014** |
| --- | --- | --- |
| Age | ✓ | ✓ |
| Sex | ✓ | ✓ |
| Ethnicity |  | ✓ |
| BMI |  | ✓ |
| Deprivation score |  | ✓ |
| Systolic blood pressure | ✓ | ✓ |
| Total cholesterol | ✓ | ✓ |
| HDL cholesterol | ✓ | ✓ |
| Family history of CHD/Stroke |  | ✓ |
| Smoking status | ✓ | ✓ |
| Treated hypertension |  | ✓ |
| Diabetes | ✓ | ✓ |
| ECG-LVH | ✓ |  |
| Atrial fibrillation |  | ✓ |
| Rheumatoid arthritis |  | ✓ |
| Chronic kidney disease |  | ✓ |

***Under-prescribing of anticoagulant drugs***

Exploratory analysis investigated the impact of using the CHA_2_DS_2_-VASc score to identify patients eligible for anticoagulants to reflect the updated 2014 atrial fibrillation guidelines. The difference between CHADS2 and CHA_2_DS_2_-VASc scores are presented in Table B. The proportion of patients prescribed aspirin rather than anticoagulants was calculated. The impact of allowing a prescription of aspirin in patients with a CHADS2 score of 1 was explored in accordance with the 2006 atrial fibrillation guidelines.

**Table B: Variables included in the CHADS2 and CHA_2_DS_2_-VASc stroke risk scores**

| **CHADS2** | | **CHA_2_DS_2_-VASc** | |
| --- | --- | --- | --- |
| **Variables** | **Score** | **Variables** | **Score** |
| Congestive heart failure | 1 | Congestive heart failure | 1 |
| Hypertension | 1 | Hypertension | 1 |
| Age ≥75 years | 1 | Age ≥75 years | 2 |
| Diabetes mellitus | 1 | Diabetes mellitus | 1 |
| Stroke or TIA | 2 | Stroke or TIA | 2 |
|  |  | Vascular disease | 1 |
|  |  | Age 65-74 years | 1 |
|  |  | Sex: female | 1 |

***Under-prescribing of antihypertensive drugs***

The proportions of strokes and TIAs not prescribed antihypertensive drugs were calculated separately for people with high blood pressure (≥160/100 mmHg) and people with moderately high blood pressure (≥140/90 mmHg) and coronary heart disease (CHD); chronic kidney disease (CKD); peripheral arterial disease (PAD); TIA (in stroke patients with prior TIA); diabetes mellitus and were aged over 40 years; or a 10-year CVD risk of ≥20%.

**Results**

***Under-prescribing of multiple prevention drugs***

Combinations of different primary prevention drugs clinically indicated are presented in Table C. Of the people who had a primary prevention drug clinically indicated, 9,953 had one drug indicated, 6,904 had two drugs indicated and 823 had three drugs indicated. For people with two drugs indicated, the most common combination was lipid lowering and antihypertensive drugs (n=5,451), followed by lipid lowering and anticoagulant drugs (n=1,389) and anticoagulant and antihypertensive drugs (n=64).

Combinations of under-prescribing for different primary prevention drugs are presented in Table D. The majority of under-prescribing occurred in people eligible for one prevention drug (n=4,887). Of people with two prevention drugs indicated, most under-prescribing occurred for one out of two of the drugs indicated. The same was true for when three prevention drugs were clinically indicated.

**Table C: Combinations of primary prevention drugs clinically indicated.**

| **Number of prevention drug classes clinically indicated** | **Prevention drugs clinically indicated** | **Number of strokes and TIAs with prevention drugs clinically indicated (n (%))** |
| --- | --- | --- |
| 0 | None | 11,363 (39.1) |
| 1 | Lipid lowering drugs | 8,365 (28.8) |
|  | Anticoagulants drugs | 918 (3.2) |
|  | Antihypertensive drugs | 670 (2.3) |
| 2 | Lipid lowering and anticoagulant drugs | 1,389 (4.8) |
|  | Lipid lowering and antihypertensive drugs | 5,451 (18.8) |
|  | Anticoagulant and antihypertensive drugs | 64 (0.2) |
| 3 | Lipid lowering, anticoagulant and antihypertensive drugs | 823 (2.8) |

**Table D: Combinations of prevention drugs under-prescribed.**

| **Prevention drugs clinically indicated** | **Number of drugs not prescribed** | **Under-prescribing** | **Number of strokes and TIAs with drugs under-prescribed (n)** |
| --- | --- | --- | --- |
| Lipid lowering drugs only | 0 | None | 4,231 |
|  | 1 | Lipid lowering drugs | 4,134 |
| Anticoagulants drugs only | 0 | None | 408 |
|  | 1 | Anticoagulant drugs | 510 |
| Antihypertensive drugs only | 0 | None | 427 |
|  | 1 | Antihypertensive drugs | 243 |
| Lipid lowering and anticoagulant drugs | 0 | None | 429 |
|  | 2 | Both drugs not prescribed | 320 |
|  | 1 | Lipid lowering drugs only | 286 |
|  |  | Anticoagulant drugs only | 354 |
| Lipid lowering and antihypertensive drugs | 0 | None | 2,369 |
|  | 2 | Both drugs not prescribed | 1,036 |
|  | 1 | Lipid lowering drugs only | 1,684 |
|  |  | Antihypertensive drugs only | 362 |
| Anticoagulant and antihypertensive drugs | 0 | None | 25 |
|  | 2 | Both drugs not prescribed | 10 |
|  | 1 | Anticoagulants drugs only | 27 |
|  |  | Anticoagulant drugs only | 2 |
| Lipid lowering, anticoagulant and antihypertensive drugs | 0 | None | 212 |
|  | 1 | Lipid lowering drugs only | 145 |
|  |  | Anticoagulant drugs only | 209 |
|  |  | Antihypertensive drugs only | 13 |
|  | 2 | Lipid lowering and anticoagulant drugs | 170 |
|  |  | Lipid lowering and antihypertensive drugs | 27 |
|  |  | Anticoagulant and antihypertensive drugs | 13 |
|  | 3 | All three drugs not prescribed | 34 |

***Under-prescribing of lipid lowering drugs***

Lowering the 10-year CVD risk threshold to ≥10% increased the number of eligible patients to 19,462, of which, 54% were not prescribed lipid lowering drugs. When QRISK2 was used to calculate CVD risk, the number of eligible patients increased to 19,253 and 53% were not prescribed lipid lowering drugs (10,237/19,253).

***Under-prescribing of anticoagulant drugs***

Use if the CHA_2_DS_2_-VASc score to calculate stroke risk increased the number of patients eligible for anticoagulant therapy by 280 and the proportion of drugs not prescribed became 50.0% (1,738/3,474). Aspirin was prescribed in 71% (1,168/1,647) of patients with anticoagulant drugs not prescribed. Allowing patients with a CHADS2 score of 1 to be prescribed aspirin or anticoagulant drugs reduced the proportion patients with anticoagulant drugs not prescribed to 40% (1,277/3,194). Aspirin was still inappropriately prescribed in 62% (798/1,277) of patients with anticoagulant drugs not prescribed under this definition (i.e. patients with CHADS2 ≥2).

***Under-prescribing of antihypertensive drugs***

For people with antihypertensive drugs were clinically indicated, 2,038 had high blood pressure (≥160/100 mmHg) and 6,272 had moderately high blood pressure (≥140/90 mmHg) with existing CVD or at high CVD risk (groups not mutually exclusive). There was under-prescribing in 27% (540/2,038) of patients with high blood pressure (≥160/100 mmHg) and 24% (1,484/6,272) with moderately high blood pressure (≥140/90 mmHg) and existing CVD or at high CVD risk (groups not mutually exclusive).

**UK estimates of under-prescribing**

Estimates in Tables E to I were calculated using data from stroke only and stroke with previous TIA from the THIN database sample population (i.e. TIA only patients were excluded). The estimated numbers of strokes prevented are based on the relative risks of 0.78 for statins,^1^ 0.68 for anticoagulants if patients were prescribed aspirin,^2^ 0.39 for anticoagulants if patients were not prescribed aspirin^3^ and 0.68 for antihypertensive drugs.^4^

**Calculations**

**Proportion of under-prescribing in THIN** =

Number of drugs not prescribed in the THIN sample / Number of strokes in the THIN sample

**Estimated number of under-prescribing in UK** =

Number of strokes per year in the UK x Proportion of under-prescribed drugs in the THIN sample

**Estimated number of strokes that could be prevented in the THIN sample** =

Number of people with drugs not prescribed in the THIN sample x RR of prevention drug(s)

**Proportion of strokes that could be prevented in the THIN sample =**

Estimated number of strokes that could be prevented in the THIN sample **/** Number of strokes in the THIN sample

**Estimated number of strokes that could be prevented in UK** =

Proportion of strokes that could be prevented in the THIN sample x Number of strokes per year in the UK

**References**

^1^ Taylor F, Huffman MD, Macedo AF, et al. Statins for the primary prevention of cardiovascular disease. The Cochrane Database of Systematic Reviews. 2013;1:Cd004816.

^2^ Aguilar MI, Hart R, Pearce LA. Oral anticoagulants versus antiplatelet therapy for preventing stroke in patients with non-valvular atrial fibrillation and no history of stroke or transient ischemic attacks. The Cochrane Database of Systematic Reviews. 2007(3):Cd006186.

^3^ Aguilar MI, Hart R. Oral anticoagulants for preventing stroke in patients with non-valvular atrial fibrillation and no previous history of stroke or transient ischemic attacks. The Cochrane Database of Systematic Reviews. 2005(3):Cd001927.

^4^ Psaty BM, Lumley T, Furberg CD, et al. Health outcomes associated with various antihypertensive therapies used as first-line agents: a network meta-analysis. JAMA. May 21 2003;289(19):2534-2544.

**Table E: Estimated number of strokes eligible for stroke prevention drugs in the UK.**

| **Age band** | **Number of strokes in the THIN sample** | | **Number of under-prescribing for one or more prevention drug in the THIN sample** | | **Proportion of under-prescribing in THIN (%)** | | **Number of strokes per year in UK** | | **Estimated number of under-presribing in UK** | |
| --- | --- | --- | --- | --- | --- | --- | --- | --- | --- | --- |
|  | Male | Female | Male | Female | Male | Female | Male | Female | Male | Female |
| <35 | 115 | 124 | 7 | 7 | 6% | 6% | 0 | 0 | 0 | 0 |
| 35-44 | 301 | 258 | 35 | 33 | 12% | 13% | 1,469 | 896 | 171 | 115 |
| 45-54 | 841 | 652 | 234 | 102 | 28% | 16% | 2,453 | 1,097 | 683 | 172 |
| 55-64 | 1,669 | 1,034 | 736 | 222 | 44% | 21% | 6,712 | 4,413 | 2,960 | 947 |
| 65-74 | 2,462 | 1,903 | 1,191 | 567 | 48% | 30% | 18,817 | 12,744 | 9,103 | 3,797 |
| 75-84 | 2,613 | 2,925 | 805 | 955 | 31% | 33% | 14,656 | 20,001 | 4,515 | 6,530 |
| ≥85 | 1,232 | 2,468 | 509 | 1,241 | 41% | 50% | 9,747 | 16,677 | 4,027 | 8,386 |
| All ages | 18,597 | | 6,644 | | 35.7% | | 109,682 | | 41,405 | |
| <85 | 14,658 | | 4,880 | | 33.3% | | 83,258 | | 28,992 | |

**Table F: Estimated number of strokes prevented in the UK: Statin, anticoagulant and antihypertensive drugs.**

| **Age band** | **Number of strokes in the THIN sample** | | **Estimated number of strokes that could be prevented in the THIN sample** | | **Proportion of strokes that could be prevented in the THIN sample** | | **Number of strokes per year in UK** | | **Estimated number of strokes that could be prevented in UK** | |
| --- | --- | --- | --- | --- | --- | --- | --- | --- | --- | --- |
|  | Male | Female | Male | Female | Male | Female | Male | Female | Male | Female |
| <35 | 115 | 124 | 2 | 2 | 1% | 1% | 0 | 0 | 0 | 0 |
| 35-44 | 301 | 258 | 10 | 9 | 3% | 3% | 1,469 | 896 | 50 | 30 |
| 45-54 | 841 | 652 | 63 | 27 | 7% | 4% | 2,453 | 1,097 | 184 | 46 |
| 55-64 | 1,669 | 1,034 | 195 | 60 | 12% | 6% | 6,712 | 4,413 | 784 | 256 |
| 65-74 | 2,462 | 1,903 | 317 | 159 | 13% | 8% | 18,817 | 12,744 | 2,426 | 1,063 |
| 75-84 | 2,613 | 2,925 | 230 | 282 | 9% | 10% | 14,656 | 20,001 | 1,288 | 1,931 |
| ≥85 | 1,232 | 2,468 | 156 | 374 | 13% | 15% | 9,747 | 16,677 | 1,237 | 2,529 |
| All ages | 18,597 | | 1,886 | | 10.1% | | 109,682 | | 11,823 | |
| <85 | 14,658 | | 1,352 | | 9.2% | | 83,258 | | 8,057 | |

**Table G: Estimated number of strokes prevented in the UK: Statin drugs only.**

| **Age band** | **Number of strokes in the THIN sample** | | **Estimated number of strokes that could be prevented in the THIN sample** | | **Proportion of strokes that could be prevented in the THIN sample** | | **Number of strokes per year in UK** | | **Estimated number of strokes that could be prevented in UK** | |
| --- | --- | --- | --- | --- | --- | --- | --- | --- | --- | --- |
|  | Male | Female | Male | Female | Male | Female | Male | Female | Male | Female |
| <35 | 115 | 124 | 1 | 1 | 1% | 1% | 0 | 0 | 0 | 0 |
| 35-44 | 301 | 258 | 8 | 7 | 3% | 3% | 1,469 | 896 | 39 | 23 |
| 45-54 | 841 | 652 | 57 | 23 | 7% | 4% | 2,453 | 1,097 | 165 | 39 |
| 55-64 | 1,669 | 1,034 | 182 | 50 | 11% | 5% | 6,712 | 4,413 | 731 | 212 |
| 65-74 | 2,462 | 1,903 | 289 | 133 | 12% | 7% | 18,817 | 12,744 | 2,208 | 889 |
| 75-84 | 2,613 | 2,925 | 167 | 188 | 6% | 6% | 14,656 | 20,001 | 939 | 1,287 |
| ≥85 | 1,232 | 2,468 | 115 | 281 | 9% | 11% | 9,747 | 16,677 | 913 | 1,899 |
| All ages | 18,597 | | 1,502 | | 8.1% | | 109,682 | | 9,343 | |
| <85 | 14,658 | | 1,103 | | 7.5% | | 83,258 | | 6,531 | |

**Table H: Estimated number of strokes prevented in the UK: Anticoagulant drugs only.**

| **Age band** | **Number of strokes in the THIN sample** | | **Estimated number of strokes that could be prevented in the THIN sample** | | **Proportion of strokes that could be prevented in the THIN sample** | | **Number of strokes per year in UK** | | **Estimated number of strokes that could be prevented in UK** | |
| --- | --- | --- | --- | --- | --- | --- | --- | --- | --- | --- |
|  | Male | Female | Male | Female | Male | Female | Male | Female | Male | Female |
| <35 | 115 | 124 | 0 | 0 | 0% | 0% | 0 | 0 | 0 | 0 |
| 35-44 | 301 | 258 | 0 | 0 | 0% | 0% | 1,469 | 896 | 0 | 0 |
| 45-54 | 841 | 652 | 1 | 1 | 0% | 0% | 2,453 | 1,097 | 4 | 3 |
| 55-64 | 1,669 | 1,034 | 7 | 3 | 0% | 0% | 6,712 | 4,413 | 28 | 14 |
| 65-74 | 2,462 | 1,903 | 20 | 21 | 1% | 1% | 18,817 | 12,744 | 154 | 140 |
| 75-84 | 2,613 | 2,925 | 70 | 102 | 3% | 3% | 14,656 | 20,001 | 394 | 698 |
| ≥85 | 1,232 | 2,468 | 58 | 151 | 5% | 6% | 9,747 | 16,677 | 460 | 1,022 |
| All ages | 18,597 | | 436 | | 2.3% | | 109,682 | | 2,918 | |
| <85 | 14,658 | | 227 | | 1.5% | | 83,258 | | 1,435 | |

**Table I: Estimated number of strokes prevented in the UK: Antihypertensive drugs only.**

| **Age band** | **Number of strokes in the THIN sample** | | **Estimated number of strokes that could be prevented in the THIN sample** | | **Proportion of strokes that could be prevented in the THIN sample** | | **Number of strokes per year in UK** | | **Estimated number of strokes that could be prevented in UK** | |
| --- | --- | --- | --- | --- | --- | --- | --- | --- | --- | --- |
|  | Male | Female | Male | Female | Male | Female | Male | Female | Male | Female |
| <35 | 115 | 124 | 0 | 1 | 0% | 1% | 0 | 0 | 0 | 0 |
| 35-44 | 301 | 258 | 6 | 3 | 2% | 1% | 1,469 | 896 | 27 | 12 |
| 45-54 | 841 | 652 | 23 | 11 | 3% | 2% | 2,453 | 1,097 | 67 | 18 |
| 55-64 | 1,669 | 1,034 | 63 | 21 | 4% | 2% | 6,712 | 4,413 | 253 | 90 |
| 65-74 | 2,462 | 1,903 | 107 | 55 | 4% | 3% | 18,817 | 12,744 | 815 | 371 |
| 75-84 | 2,613 | 2,925 | 55 | 64 | 2% | 2% | 14,656 | 20,001 | 311 | 435 |
| ≥85 | 1,232 | 2,468 | 36 | 68 | 3% | 3% | 9,747 | 16,677 | 282 | 458 |
| All ages | 18,597 | | 512 | | 2.8% | | 10,9682 | | 3,137 | |
| <85 | 14,658 | | 408 | | 2.8% | | 83,258 | | 2,398 | |
