# Supplementary material for: Under-prescribing of Prevention Drugs and Primary Prevention of Stroke and Transient Ischaemic Attack in UK General Practice: A Retrospective Analysis
Source: PLoS Med. 2016 Nov 15;13(11):e1002169. doi: 10.1371/journal.pmed.1002169 (PMC5112771; doi:10.1371/journal.pmed.1002169)
Supplement: S1 Fig — (DOCX) [file pmed.1002169.s002.docx]

**S1 Figure: Crude incidence of stroke and transient ischaemic attack (TIA) recorded in The Health Improvement Network (THIN) database.**
