# Supplementary material for: Under-prescribing of Prevention Drugs and Primary Prevention of Stroke and Transient Ischaemic Attack in UK General Practice: A Retrospective Analysis
Source: PLoS Med. 2016 Nov 15;13(11):e1002169. doi: 10.1371/journal.pmed.1002169 (PMC5112771; doi:10.1371/journal.pmed.1002169)

**S2 Figure: Summary of under-prescribing of stroke prevention drugs and exception reporting (dugs declined or contraindicated).**


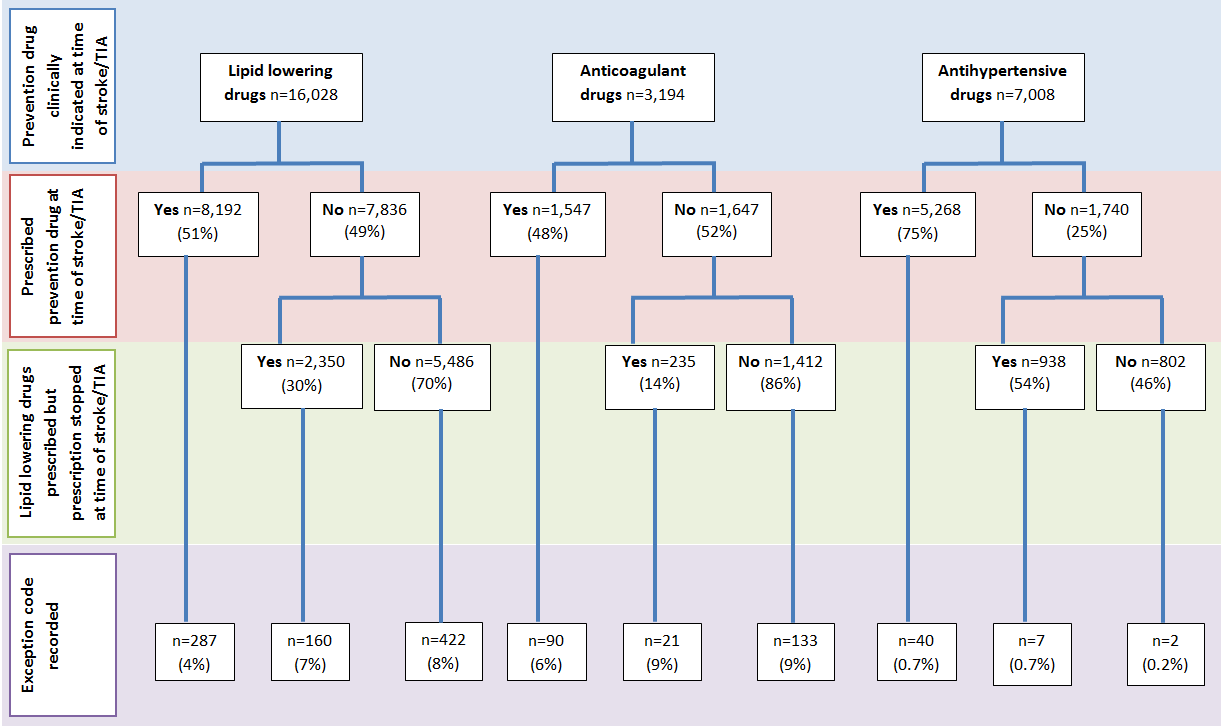

Supplement: S2 Fig — (DOCX) [file pmed.1002169.s003.docx]
