# Supplementary material for: Under-prescribing of Prevention Drugs and Primary Prevention of Stroke and Transient Ischaemic Attack in UK General Practice: A Retrospective Analysis
Source: PLoS Med. 2016 Nov 15;13(11):e1002169. doi: 10.1371/journal.pmed.1002169 (PMC5112771; doi:10.1371/journal.pmed.1002169)
Supplement: S1 Table — (DOCX) [file pmed.1002169.s006.docx]

**S1 Table: Values outside clinically plausible ranges which were excluded.**

| **Variable** | **Cut-off range** |
| --- | --- |
| Height (m) | 1 - 2.5 |
| Weight (kg) | 35 - 200 |
| Body mass index (units) | 10 - 60 |
| Systolic blood pressure (mmHg) | 60 – 260 |
| Diastolic blood pressure (mmHg) | 40 - 160 |
| Total cholesterol (mm/L) | 1 - 12 |
| High-density lipoprotein (HDL) cholesterol (mm/L) | 0.1 - 12 |
