# Supplementary material for: Under-prescribing of Prevention Drugs and Primary Prevention of Stroke and Transient Ischaemic Attack in UK General Practice: A Retrospective Analysis
Source: PLoS Med. 2016 Nov 15;13(11):e1002169. doi: 10.1371/journal.pmed.1002169 (PMC5112771; doi:10.1371/journal.pmed.1002169)
Supplement: S2 Table — (DOCX) [file pmed.1002169.s007.docx]

**S2 Table: Number of general practices contributing data to the study between 2000 and 2013.**

| **Year** | 2000 | 2001 | 2002 | 2003 | 2004 | 2005 | 2006 | 2007 | 2008 | 2009 | 2010 | 2011 | 2012 | 2013 |
| --- | --- | --- | --- | --- | --- | --- | --- | --- | --- | --- | --- | --- | --- | --- |
| **Number of general practices** | 6 | 9 | 13 | 14 | 14 | 12 | 150 | 195 | 319 | 428 | 431 | 436 | 490 | 482 |
